# Enhancing Leaf Area Index Estimation for Maize with Tower-Based Multi-Angular Spectral Observations

**DOI:** 10.3390/s23229121

**Published:** 2023-11-11

**Authors:** Lieshen Yan, Xinjie Liu, Xia Jing, Liying Geng, Tao Che, Liangyun Liu

**Affiliations:** 1 College of Geomatics, Xi’an University of Science and Technology, Xi’an 710054, China; 21210226046@stu.xust.edu.cn (L.Y.);; 2 International Research Center of Big Data for Sustainable Development Goals, Beijing 100094, China; liuxj@radi.ac.cn; 3 Aerospace Information Research Institute, Chinese Academy of Sciences, Beijing 100094, China; 4 Heihe Remote Sensing Experimental Research Station, Key Laboratory of Remote Sensing of Gansu Province, Northwest Institute of Eco-Environment and Resources, Chinese Academy of Sciences, Lanzhou 730000, China

**Keywords:** multi-angular spectral observation, leaf area index (LAI), tower-based platform, MAVI

## Abstract

The leaf area index (LAI) played a crucial role in ecological, hydrological, and climate models. The normalized difference vegetation index (NDVI) has been a widely used tool for LAI estimation. However, the NDVI quickly saturates in dense vegetation and is susceptible to soil background interference in sparse vegetation. We proposed a multi-angular NDVI (MAVI) to enhance LAI estimation using tower-based multi-angular observations, aiming to minimize the interference of soil background and saturation effects. Our methodology involved collecting continuous tower-based multi-angular reflectance and the LAI over a three-year period in maize cropland. Then we proposed the MAVI based on an analysis of how canopy reflectance varies with solar zenith angle (SZA). Finally, we quantitatively evaluated the MAVI’s performance in LAI retrieval by comparing it to eight other vegetation indices (VIs). Statistical tests revealed that the MAVI exhibited an improved curvilinear relationship with the LAI when the NDVI is corrected using multi-angular observations (R^2^ = 0.945, RMSE = 0.345, rRMSE = 0.147). Furthermore, the MAVI-based model effectively mitigated soil background effects in sparse vegetation (R^2^ = 0.934, RMSE = 0.155, rRMSE = 0.157). Our findings demonstrated the utility of tower-based multi-angular spectral observations in LAI retrieval, having the potential to provide continuous data for validating space-borne LAI products. This research significantly expanded the potential applications of multi-angular observations.

## 1. Introduction

The leaf area index (LAI) stands as a paramount structural parameter in terrestrial eco-systems, intimately intertwined with plant transpiration, photosynthesis, and surface net primary productivity [1,2,3]. Its swift and precise estimation holds immense importance for scrutinizing global carbon cycling and climate changes [4].

Remote sensing technology enables extensive ground observations, furnishing a potent tool for ascertaining LAI across regional vegetation. Researchers often gauge LAI from remote sensing data by relying on empirical connections between the LAI and vegetation indice (VI), notably the NDVI [5,6,7]. The computation of the NDVI is straightforward, and it exhibits a robust correlation with vegetation health, rendering it widely adopted for monitoring vegetation growth, including LAI estimation [8,9,10]. Nonetheless, two significant challenges loom when employing the NDVI for LAI retrieval. Firstly, the NDVI proves highly sensitive to variations in soil background brightness in sparsely vegetated areas [11]. Secondly, the LAI–NDVI correlation is substantially influenced by saturation effects, making accurate LAI predictions from NDVI quite challenging [12,13,14].

To circumvent the influence of soil background and saturation effects, recent research in the past two decades has proposed VIs that incorporate nadir reflectance at shortwave infrared (SWIR) bands [2,15,16]. Meanwhile, the wide dynamic range vegetation index (WDRVI) emerged to mitigate the saturation in LAI retrieval [17]. Nevertheless, VIs calculated using single angular reflectance often fall short in accurately characterizing vegetation information in many scenarios. The reason is that the three-dimensional structure information of vegetation cannot be well described by the mono-angular measurements [18,19,20].

Multi-angular observations hold the potential to furnish information regarding vegetation canopy structure and, consequently, can enhance the precision of LAI retrieval [21,22,23]. Previous studies have put forth empirical bidirectional reflectance distribution function (BRDF) models that incorporate the reflectance of hotspots and darkspots, offering superior predictability of vegetation parameters compared to traditional VIs [24,25]. Lacaze et al. introduced a directional index (HDS) utilizing hotspot–darkspot information, effectively reflecting the geometric structure of vegetation [26]. Chen et al. proposed a normalized difference between hotspot and darkspot index (NDHD), proven to enhance LAI retrieval accuracy [16,21]. Researchers also discovered that the normalized hotspot-signature vegetation index (NHVI), calculated by multiplying the NDVI and the HDS, could mitigate the NDVI’s insensitivity to LAI in dense vegetation [5]. Wu et al. introduced a hotspot–darkspot difference index (HDDI) founded on multi-angular optical remote sensing, better suited for LAI estimation than the NDVI and the EVI [27]. Pocewicz et al. computed two anisotropic versions of the NDVI, adjusted for nadir (NDVI_HS_) and hotspot–darkspot (NDVI_HD_) for LAI retrieval, affirming the critical importance of multi-angular observations [28]. These models possess the potential to advance LAI prediction accuracy by incorporating additional information [28,29,30].

Tower-based platform remote sensing, a crucial facet of remote sensing for acquiring vegetation information, serves as a “bridge” linking flux stations and satellite remote sensing, exhibiting substantial potential across ecological function assessment, carbon cycling, crop monitoring, and other domains [31]. In recent years, the tower-based platform spectral automatic observation network has made significant strides. For example, an optical network, EUROSPEC, was used to conduct long-term ground-based optical measurements at the representative EC towers in the European Union (EU) [32,33]. In addition, Zhang et al. also established a network (ChinaSpec) for long-term ground-based measurements of solar-induced fluorescence in China [34], which offers long-term spectral observation data for various ecosystem types.

This system of tower-based platform remote sensing collects vegetation spectra at different solar zenith angles (SZAs) from a fixed viewing perspective, allowing for canopy reflectance calculations from multiple angles by combining descending irradiance. Consequently, fixed station multi-angular observations yield ample information for high-precision LAI retrieval, and the estimated values of LAI hold promise as sources of high-quality LAI validation data for satellite remote sensing [31,34]. Nevertheless, while numerous theories of multi-angular remote sensing have focused on utilizing hotspot and darkspot information, few studies have explored the feasibility of tower-based multi-angular observation for LAI estimation.

In this study, three years of field measurements were collected to develop an improved LAI estimation method derived from tower-based multi-angular spectral observations. Therefore, the objectives of this paper are (1) to scrutinize the impact of soil background and LAI on vegetation canopy reflectance under various SZAs; (2) to formulate an efficient yet uncomplicated method (MAVI) for mitigating interference of soil and saturation in VI-based models; and (3) to assess the performance of the enhanced MAVI-based model against traditional VI-based models in terms of applicability and accuracy in predicting LAI.

## 2. Materials and Methods

### 2.1. Tower-Based Multi-Angular Reflectance Measurements

Multi-angular spectral observations were conducted at the DaMan (DM) site, situated in the Daman irrigation area in the middle reaches of the Heihe River Basin, Zhangye City, Gansu Province, China (38°51′20″ N, 100°22′20″ E). The study area exemplifies a typical oasis with flat, open terrain and uniform vegetation. The DM site experiences a temperate semi-arid continental climate with an average annual temperature of 8 °C. The primary crop grown in this region is maize, cultivated without specific fertilization control, typically sown in mid-May and harvested in mid-September [31,35,36].

To acquire multi-angular spectral observation reflectance, a long-term spectral observation system was established on a tower-based platform, positioned approximately 25 m above the ground [34,37]. The comprehensive observation system comprises three key components: an Ocean Optics QE65 Pro spectrometer (Ocean Optics, Inc., Dunedin, FL, USA), a computer control system, and a temperature control system. The QE65 Pro spectrometer boasts a spectral resolution of 0.34 nm, a sampling interval of 0.155 nm, a spectral range spanning 650–805 nm, and a signal-to-noise ratio (SNR) exceeding 1000 [37]. The computer control system encompasses a computer for storing spectral data and instrument operation status, along with software responsible for managing instrument functionality [38].

To ensure the spectrometer’s continuous operation under variable field conditions, it is enclosed within a temperature-controlled chamber, and precise temperature regulation is maintained, with temperature variations within 25 ± 1 °C. A fiber optic probe (field of view (FOV) of 180°), connected to a cosine corrector (CC3-3-UV-S, Ocean Optics, Inc., Dunedin, FL, USA), and a bare optical fiber probe (FOV of 25°) are connected to the spectrometer via a Y-shaped bifurcated optical fiber (CPATCH, Ocean Optics, Inc., Dunedin, FL, USA) [37]. The former captures downwelling incident irradiance in a vertical direction, while the latter is angled downward (viewing zenith angle (VZA) of 25°) to measure upwelling radiance. The underlying surface around the site is a uniform maize field, and the sensor is high enough to cover a representative field of view.

The automatic observation system operates in a “sandwich” configuration [39], wherein solar irradiance, canopy-reflected radiance, and solar irradiance are sequentially measured by controlling electronic switches. The time delay between upwelling radiance and downwelling irradiance measurements in the observation system is relatively low (a few seconds). To mitigate the impact of time delay, the average of the two solar irradiance measurements is computed. Furthermore, the control system automatically optimizes the integration time based on lighting conditions, aiming to strike an optimal balance between SNR and observation speed [38]. Different to traditional multi-angular observations, this study made use of varying SZAs but a fixed VZA, which means that the sensors can be fixed, and can be amounted on any ground or tower-based platforms.

**Figure 1:**
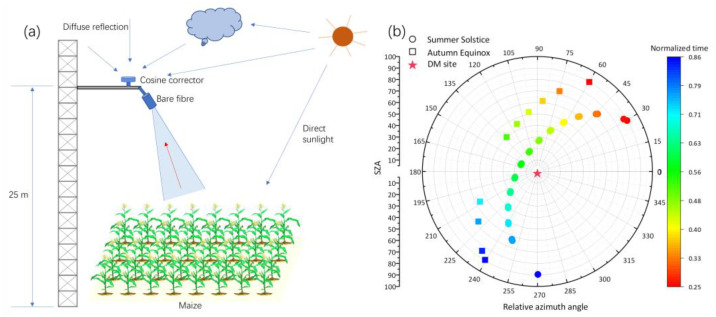
(**a**) Illustration of the tower-based observation system; (**b**) variations in SZA and relative azimuth angle (RAA) over time at the site (measurements from 2018; summer solstice: DOY 172; autumn equinox: DOY 266).

**Figure 2:**
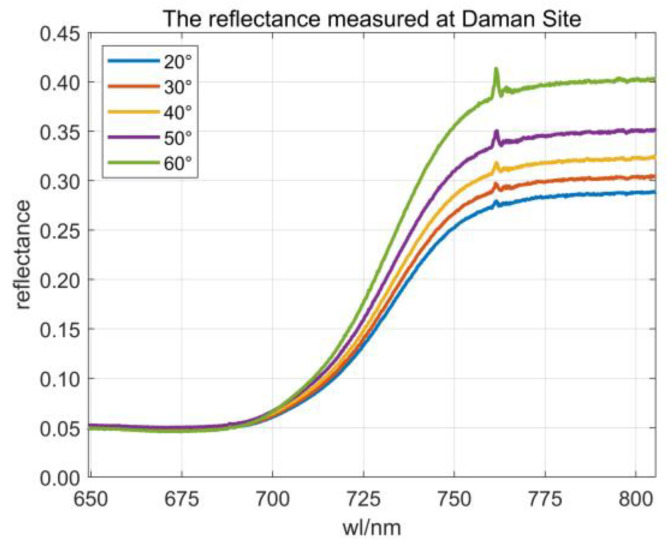
Measured reflectance in the wavelength range of 650–805 nm (angles of the figure represent SZAs), and the observation date is 21 June 2018.

Figure 1a illustrates a schematic diagram of the continuous observation system, which provided a clear view of the experimental platform’s structure; Figure 1b shows the polar diagram of the changes in SZA and RAA over time in a day. Throughout the day, the SZA undergoes variations, with the automatic observation system continuously monitoring the same vegetation at a fixed location, capturing reflectance at various SZAs (Figure 2). This study specifically selected multi-angular reflectance data from morning to noon daily for subsequent experiments. To ensure the validity of experimental data, the SZAs of measured reflectance were confined to within 60°. Moreover, changes in lighting conditions were considered in the LAI retrieval process. Based on the clear sky index (CI), this study filtered the observed data to include only clear-sky conditions (CI > 0.5) for subsequent experiments [40].

### 2.2. Leaf Area Index Measurement

**Figure 3:**
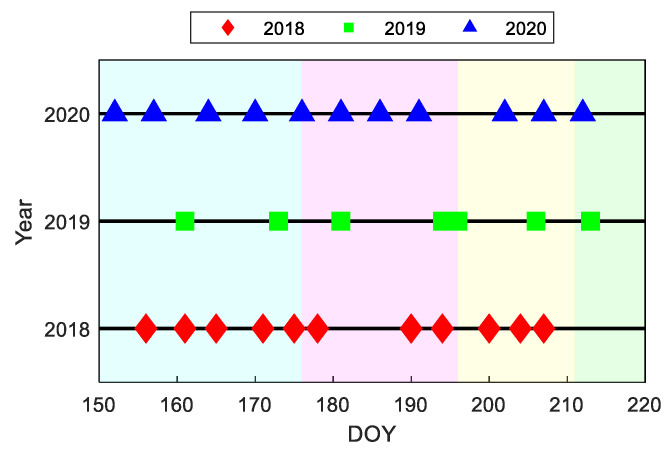
Temporal distribution of the measurements, where DOY represents a day of the year (the shadows in the figure represent the emergence period, elongation period, big trumpet period, and tasseling period in sequence).

To validate LAI retrieval accuracy based on tower-based multi-angular observations, a LAI 2200 instrument (LI-COR, Inc., Lincoln, NE, USA) was employed to measure the LAI near the DM site in 2018, 2019, and 2020. To avoid the influence of solar angle and shadow on LAI measurement, we performed LAI measurements in the early morning or late afternoon. During field observations, each canopy upward radiance measurement corresponds to four canopy transmitted radiance measurements. This operation process was repeated twice, and the average of the two measurements yielded a LAI. Three 10 × 10 m quadrats near the site were selected for measurement, and the sample information is shown in Table 1. Two LAI measurements were conducted in each quadrat, so a total of six LAI measurements were obtained. To ensure data accuracy, measurements outside the range of μ ± σ (where μ and σ are the mean and standard deviation, respectively) were excluded from the data processing, and the remaining data were averaged to determine the LAI for a specific day. To capture the LAI at different stages of vegetation growth, LAI measurements were conducted approximately every 5 days, as indicated in Figure 3. This study accumulated 29 days of measurements at the DM site during the growing seasons of 2018, 2019, and 2020 [36,41,42,43,44].

### 2.3. Simulated Datasets

In order to quantitatively analyze the BRDF effect of the reflectance, the PROSAIL model (PROSAIL_D) was employed to simulate crop spectral reflectance. PROSAIL comprised the PROSPECT model and the SAIL model. The PROSPECT model is a leaf optical model developed on the basis of Allen’s flat plate model [45,46] assuming that the leaves are composed of N layers of isotropic layers, which are separated by N-1 layers of air gaps. The upper layer of the first layer is the leaf epidermis, and the propagation of light on the leaf surface exhibits anisotropy, i.e., non-isotropy. But inside the leaves, the light propagation is considered to be isotropic.

The PROSPECT model is capable of simulating leaf reflectance within a wavelength range of 400–2500 nm. This model relies on five structural and biochemical parameters: leaf chlorophyll a and b content (C_ab_), leaf carotenoid content (C_ar_), equivalent water thickness (C_w_), leaf dry matter content (C_m_) and leaf structure coefficient (N). The leaf spectral data generated by the PROSPECT model serve as input for the SAIL canopy radiation model. The SAIL model treats vegetation as a mixed medium and assumes uniform leaf inclination distribution. Specific parameters are input into the SAIL model to simulate the bidirectional reflectance of the vegetation canopy. Eight vegetation parameters are needed for the SAIL model, which include LAI, leaf inclination angle distribution (LAD), hotspot factor (hspot), soil brightness coefficient (Ps), sky scattered light ratio (Sky1), SZA, VZA, and RAA [47]. In this study, the maize under investigation exhibits negligible variations in plant height within the same growth stage, resulting in a uniformly distributed plant canopy. Consequently, the PROSAIL model is suitable for simulating maize canopy reflectance [48,49,50,51]. The parameter settings of PROSAIL model are shown in Table 2.

### 2.4. Accuracy Assessment and Sensitivity Analysis Indices

Statistical criteria, including the coefficient of determination (R^2^) [58], the root mean square error (RMSE), and the relative root mean square error (rRMSE) were used to evaluate the performances of models in estimating LAI [59,60].

(1) $$R^{2}=1-\frac{\sum_{i=1}^{n}{\left(y_{i}-{\hat{y}}_{i}\right)}^{2}}{\sum_{i=1}^{n}{\left(y_{i}-{\bar{y}}_{i}\right)}^{2}}$$

(2) $$RMSE=\sqrt{\frac{1}{n}\sum_{i=1}^{n}{\left(y_{i}-{\hat{y}}_{i}\right)}^{2}}$$

(3) $$rRMSE=\frac{RMSE}{{\bar{y}}_{i}}$$

where $y_{i}$ and ${\hat{y}}_{i}$ are the observed and modeled values of sample i, respectively, and ${\bar{y}}_{i}$ is the mean of the observed values.

In addition, two indicators were used for parameter sensitivity analysis. Firstly, the coefficient of variation (CV) was used to evaluate the sensitivity of a particular VI/reflectance to parameter v (such as the LAI) [61].

(4) $$CV=\frac{\sigma_{v}}{\mu_{v}}$$

where $\sigma_{v}$ represents the standard deviation and $\mu_{v}$ represents the mean. A higher CV indicates that VI/reflectance is more sensitive to variations in the specific parameter v. The second indicator is the saturation point (SP), which is the starting point of each VI without responding to the increase in LAI, and which is used to evaluate the ability of a vegetation index to monitor the LAI [62]. The SP is calculated as the point where the absolute value of the first derivative to the LAI is less than a defined threshold (0.03 in this paper).

### 2.5. Developement of the MAVI

**Figure 4:**
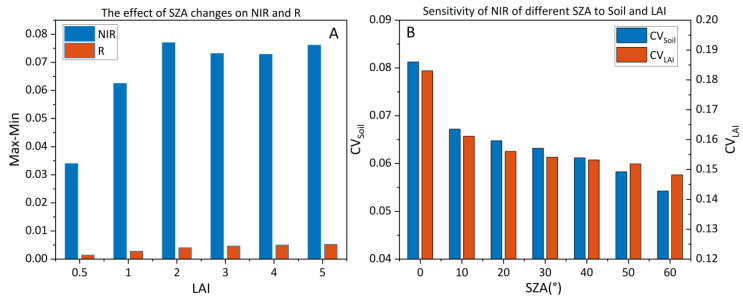
(**A**) Variation range of NIR and R with SZAs in different vegetation densities based on simulated data. The SZA is set to 0°–60°, and Max−Min represents the maximum value minus the minimum value of the reflectance. (**B**) The CV of NIR with different soil or LAI, where the soil brightness is set to 0–1 and the LAI is set to 0–5 (simulated data).

The multi-angular reflectance of the tower-based platform includes red (R), red-edge and near-infrared (NIR) bands. However, the red-edge band is very sensitive to the variations in LCC, and the introduction of a red-edge band will increase the uncertainty of the empirical relationship between the VI and the LAI [63]. Therefore, we focus on studying the BRDF effect of R (680 nm) and NIR (800 nm). To assess the impact of changes in the SZA on NIR and red bands reflectance, we varied the SZA from 0° to 60° and computed the difference between the maximum and minimum reflectance values for NIR and R. As shown in Figure 4A, the SZA has a significantly greater effect on NIR than on R. Consequently, subsequent research focused on the influence of the SZA on NIR. We simulated NIR under various soil brightness and LAI conditions across multiple SZAs. By calculating the CV for NIR with specific parameters (soil, LAI), we quantitatively evaluated the sensitivity of NIR at different SZAs to these parameters. Figure 4B demonstrates that with increasing SZA, NIR becomes less sensitive to variations in soil background and LAI. This study obtained multi-angular reflectance (measurements) within an SZA range of 20° to 60°, and, hence, subsequent research will operate within this angular range.

**Figure 5:**
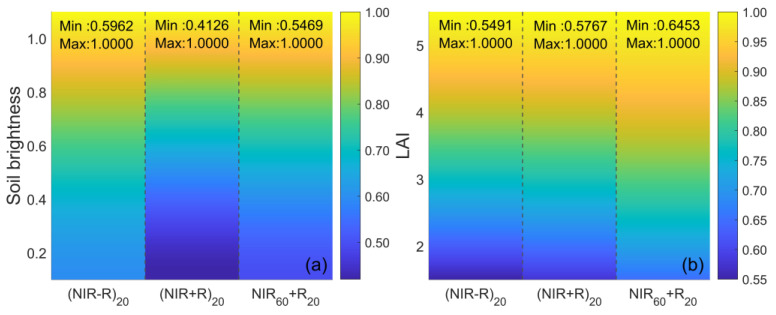
Changes in NIR − R and NIR + R with (**a**) soil brightness and (**b**) LAI, where the subscripts of reflectance represent the SZA, soil brightness is set to 0–1, and LAI is set to 2–5 (simulated data).

The NDVI is highly sensitive to changes in soil brightness and exhibits saturation effects in LAI retrieval [5]. Therefore, this study simulated (NIR−R) and (NIR + R) under different soil and LAI conditions, aiming to optimize the NDVI. As illustrated in Figure 5, (NIR−R)_20_ (the subscript indicates a specific SZA) responds less to soil variations compared to (NIR + R)_20_, and the responses of (NIR−R)_20_ and (NIR + R)_20_ to the LAI are nearly identical. This characteristic in response renders the NDVI insensitive to the LAI while remaining sensitive to soil background in LAI retrieval. To address this issue, this study capitalized on the inverse relationship between SZAs and NIR sensitivity to vegetation parameters (soil brightness, LAI), and adjusted the SZA for NIR in the NDVI denominator, introducing the concept of MAVI.

(5) $$MAVI=\frac{{NIR}_{1}-R_{1}}{{NIR}_{2}+R_{1}}$$

Among them, ${NIR}_{1}$ and $R_{1}$ represent reflectance with a small SZA, and ${NIR}_{2}$ represents reflectance with a large SZA. In the experimental phase, field-measured reflectance data were collected with SZAs ranging from 20° to 60° to establish MAVI, with SZA1 set to 20° and SZA2 to 60°.

Figure 5 shows that the sensitivity of NIR_60_ + R_20_ to soil brightness is lower than that of (NIR + R)_20_, and is roughly equivalent to (NIR − R)_20_. Additionally, the sensitivity of NIR_60_ + R_20_ to the LAI is lower than that of (NIR + R)_20_ and (NIR − R)_20_. Therefore, this modification has the ability to reduce NDVI’s sensitivity to soil brightness while enhancing its sensitivity to the LAI.

### 2.6. Other Vegetation Indices for Comparison

To evaluate the performance of the MAVI, it is compared with eight other vegetation indices, including the NDVI, the NDRE, the RVI, the DVI, the MSR, the EVI2, the SAVI, and the MSAVI, as listed in Table 3. The NDVI is one of the most widely used vegetation indices in remote sensing [64]. The NDRE is an improved version of the NDVI known for its excellent LAI prediction capability [65]. The RVI and the DVI are straightforward indices commonly used for vegetation parameter retrieval due to their simplicity [66,67]. The MSR and the EVI2 have been reported to overcome the saturation issues encountered by the NDVI [68,69], while the SAVI and the MSAVI effectively mitigated the influence of soil background on vegetation parameter retrieval [70,71]. All the VIs in this study, except for MAVI, were computed using reflectance data with an SZA of 20°.

## 3. Results

### 3.1. Sensitivity of Different VIs to Soil Background

**Figure 6:**
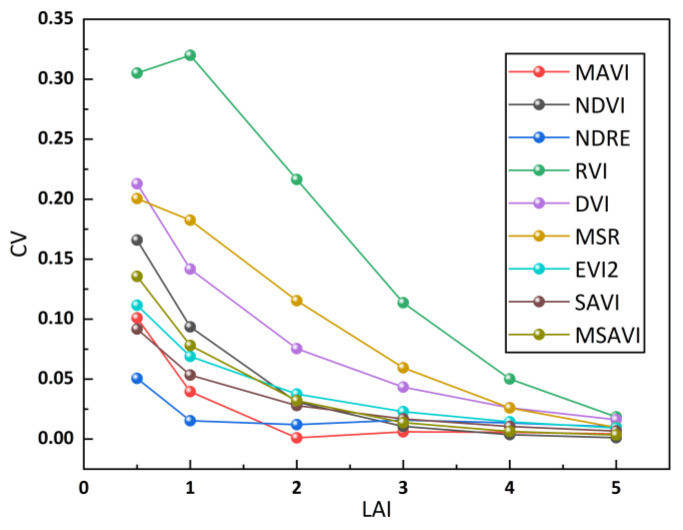
Sensitivity analysis of changes in VIs (introduced in Table 3) with soil brightness under different vegetation densities based on simulated data.

In this section, we introduced varying soil reflectance into the PROSAIL model to generate a simulated dataset and calculated nine vegetation indices based on these simulated data. We employed the CV to quantitatively assess the impact of soil background reflectance on these VIs. Figure 6 illustrates that the RVI, the MSR, and the DVI exhibit significantly higher CV values compared to the other VIs, with the RVI having the highest CV across all scenarios. This finding highlights their sensitivity to changes in soil background, making it challenging to establish robust VI–LAI empirical relationships. When the LAI is less than 2, the relatively large CV for the NDVI suggests its difficulty in disregarding the influence of soil reflectance in sparse vegetation. To address this issue, numerous improved vegetation indices, such as the EVI2, the SAVI, and the MSAVI, have been developed. It can be seen from Figure 6 that the CVs of the MSAVI, the EVI2, the and SAVI decrease in turn, indicating that the improved vegetation indices can effectively reduce the effect of soil reflectance on VIs, but there is still room for improving. In Figure 6, the NDRE and the MAVI consistently achieve the lowest CV values, signifying their effectiveness in resisting soil effects. The NDRE performs exceptionally well in the early stages of vegetation growth, whereas the MAVI excels in the later stages. However, the overall difference between them is not substantial. This outcome suggests that the MAVI, combined with multi-angular reflectance, effectively mitigated the influence of background reflectance on the vegetation index.

### 3.2. Sensitivity of Different VIs to LAI and Leaf Chlorophyll Content (LCC)

**Figure 7:**
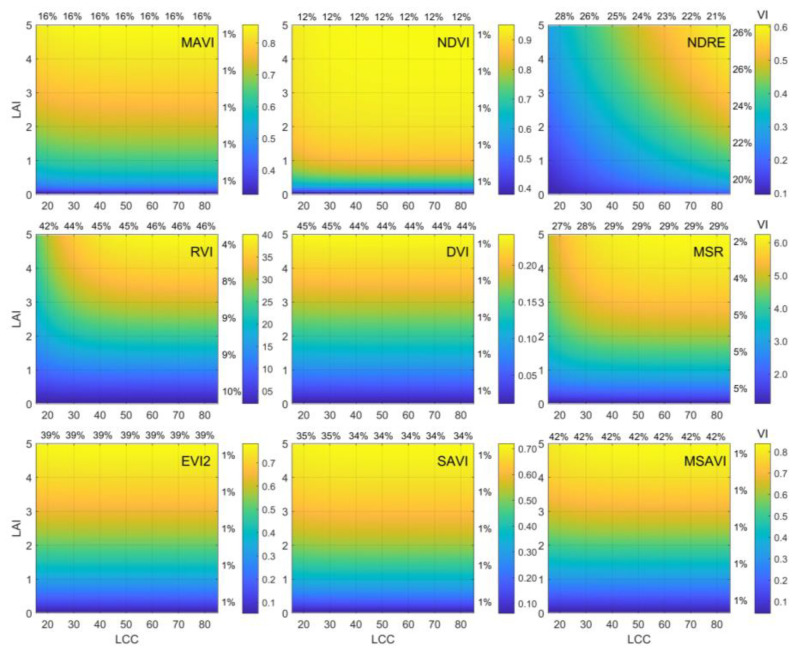
Sensitivity analysis of VIs (introduced in Table 3) to LAI and LCC based on simulated data. The top and right numbers indicate the CV of VIs to LAI and LCC at the specific value range, respectively.

An ideal vegetation index used for LAI retrieval should be sensitive to the LAI while remaining insensitive to LCC. Figure 7 demonstrates the impact of different LAI and LCC on vegetation indices. First, it is evident that, except for the NDRE, the RVI, and the MSR, all other VIs exhibit minimal CV values with changes in the LCC (CV = 1%), indicating their insensitivity to LCC variations. Simultaneously, this result implies that, compared to the NDVI, the MAVI not only eliminates the influence of soil background but also retains the NDVI’s advantage of insensitivity to LCC changes. Furthermore, Figure 7 reveals that the NDVI exhibits a CV of only 12% with LAI variations, significantly lower than other VIs. This finding suggests that the NDVI struggles to capture LAI changes effectively. Additionally, Figure 7 illustrates that the MAVI’s CV with different LAI is 16%, which is 33% higher than that of the NDVI, indicating that the MAVI is more sensitive to the LAI than the NDVI. This result demonstrates that the MAVI effectively eliminates saturation effects while remaining insensitive to the LCC.

**Figure 8:**
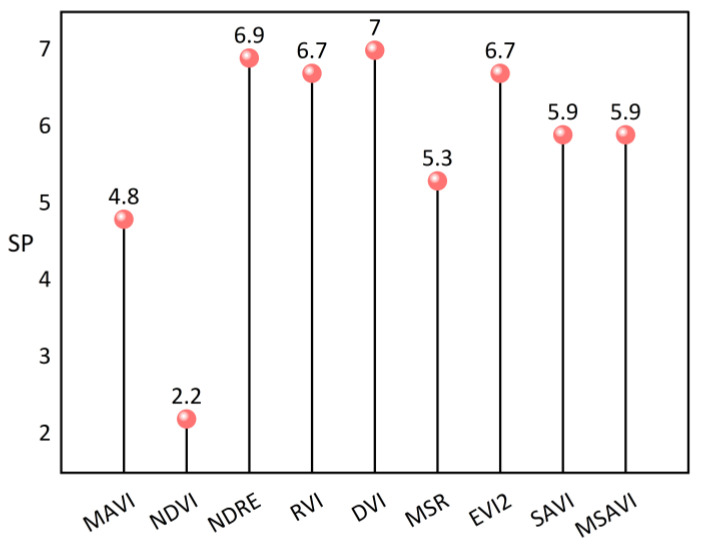
Saturation point (SP) analysis of LAI for different VIs (introduced in Table 3) based on simulated data.

To further quantitatively describe VIs’ ability to monitor the LAI, we employed the saturation point (SP) to evaluate the ability of the vegetation index to monitor the LAI. Figure 8 reveals that the DVI is the most sensitive VI to LAI changes, with the DVI, the NDRE, the RVI, the EVI2, the SAVI, and the MSAVI generally exhibiting higher SP values than other indices. In comparison to these indices, the MSR and the MAVI show slightly weaker sensitivity to the LAI, with SPs occurring at 5.3 and 4.8, respectively. Among all the studied VIs, the NDVI displays the lowest sensitivity to the LAI, implying that it struggles to monitor significant LAI changes when the LAI exceeds 2.2. In contrast, the MAVI’s SP is more than double that of the NDVI, indicating that multi-angular observation helps alleviate the NDVI’s saturation effect. Although the MAVI’s SP for LAI changes is lower than that of most vegetation indices, the measured LAI dataset suggests that maize LAI around the site typically falls below 4.5. As such, the MAVI can be effectively employed for tower-based LAI retrieval.

### 3.3. Sensitivity of Different VIs to Other Vegetation Parameters

**Figure 9:**
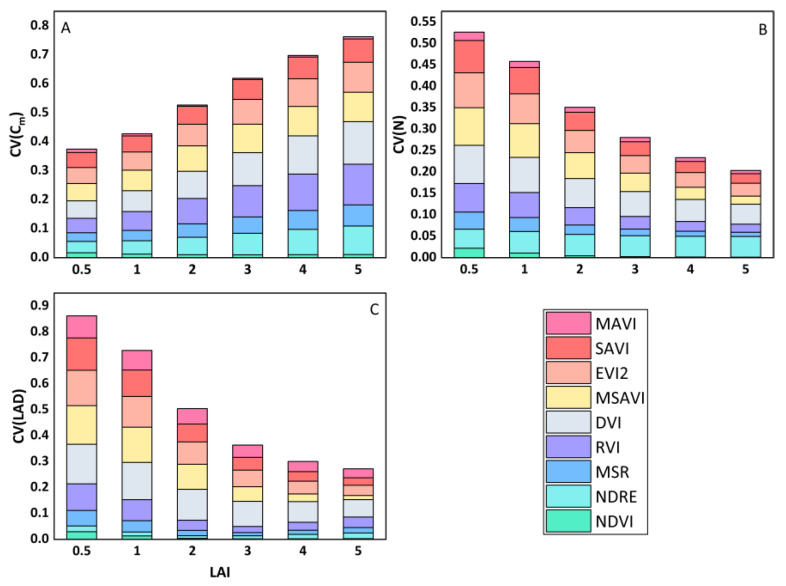
Sensitivity analysis of VI to important parameters (**A**) N, (**B**) C_m_, and (**C**) LAD based on simulated data. A longer color bar indicates that the corresponding VI (introduced in Table 3) is more sensitive to this parameter and vice versa.

In this section, we investigated how various vegetation indices respond to several key parameters (N, Cm, and LAD) using simulated data. The results are presented in Figure 9. It is evident from Figure 9 that, except for the NDVI and the MAVI, most VIs are highly sensitive to the studied parameters, including improved vegetation indices like the SAVI and the EVI2, etc. This outcome suggests that achieving low sensitivity for important vegetation parameters while enhancing LAI retrieval accuracy is challenging for most VIs. However, the MAVI and the NDVI remain largely unaffected by changes in C_m_ and N at different vegetation growth stages. This underscores that introducing multi-angular reflectance can effectively reduce the impact of soil reflectance on VI and enhance the correlation between the VI and the LAI while maintaining the VI’s insensitivity to changes in critical parameters. From this perspective, the MAVI appears more suitable for LAI estimation than most other VIs. Moreover, the MAVI is not significantly affected by LAD in dense vegetation, while it is more sensitive to changes in LAD in sparse vegetation. Although the MAVI changed with the variation in LAD, this fluctuation is not significant compared to other VIs.

### 3.4. Performance of VI-Based LAI Estimation

**Figure 10:**
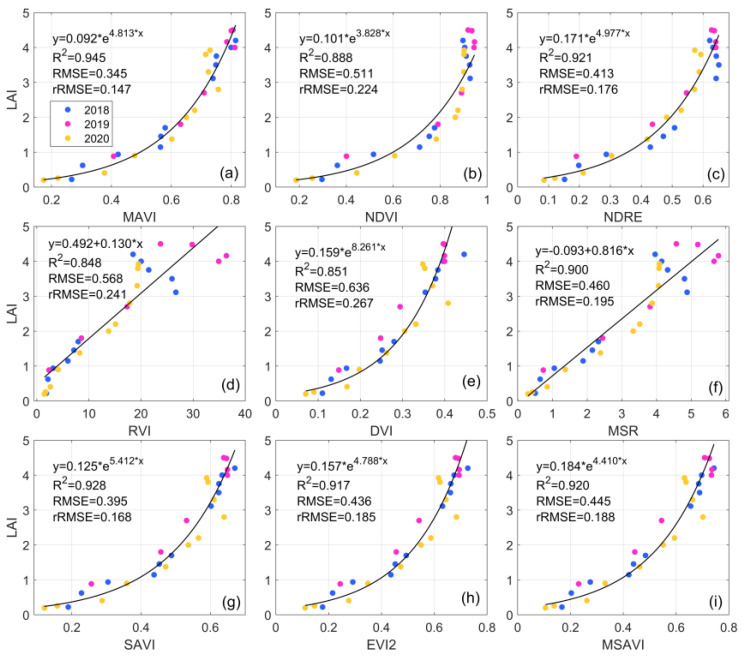
Relationship between (**a**) MAVI, (**b**) NDVI, (**c**) NDRE, (**d**) RVI, (**e**) DVI, (**f**) MSR, (**g**) SAVI, (**h**) EVI2, (**i**) MSAVI and measured LAI at the DM site (The VIs are introduced in Table 3, and the blue, red and yellow dots represent the years 2018, 2019 and 2020, respectively).

Figure 10 shows the scatter plot of the correlation between VI and measured LAI. The colors of the dots represent the years of observational data, which are 2018, 2019, and 2020, respectively. It is evident from the figure that the RVI and the MSR exhibit a linear relationship with the LAI, while the other VIs demonstrate an exponential relationship with the LAI. Among the nine estimation models, the MAVI exhibits the strongest correlation with the LAI and achieves the highest accuracy in LAI retrieval (R^2^ = 0.945, RMSE = 0.345, rRMSE = 0.147), followed by the SAVI (R^2^ = 0.928, RMSE = 0.395, rRMSE = 0.168). The RVI and the DVI have the lowest prediction accuracy for the LAI (R^2^ = 0.848, 0.851, RMSE = 0.568, 0.636, rRMSE = 0.241, 0.267). It is apparent from the figure that the fitting relationship between the NDVI and the LAI is poor in dense vegetation areas (LAI > 2), and the scattered points in sparse vegetation areas (LAI ≤ 2) do not align well with the fitting curve. This may be attributed to the influence of soil background and saturation effects on the NDVI, resulting in reduced LAI prediction accuracy. The fitting accuracy of the MAVI and the LAI is significantly superior to that of the NDVI, indicating that the MAVI, combined with multi-angular reflectance, is more suitable for tower-based LAI retrieval than the NDVI. Additionally, the results reveal that the NDRE, the DVI, the RVI, and the MSR struggle to track changes in the LAI effectively in later stages of vegetation growth, likely due to interference from other vegetation parameters in the VI–LAI empirical relationship. The SAVI, the EVI2, the and MSAVI have been proven to improve the retrieval accuracy of LAI. Similar results were obtained in this study, and the accuracy of LAI retrieval is better than that of the traditional NDVI, RVI, DVI, etc. Nevertheless, overall, the MAVI demonstrates the best predictive accuracy for LAI.

**Figure 11:**
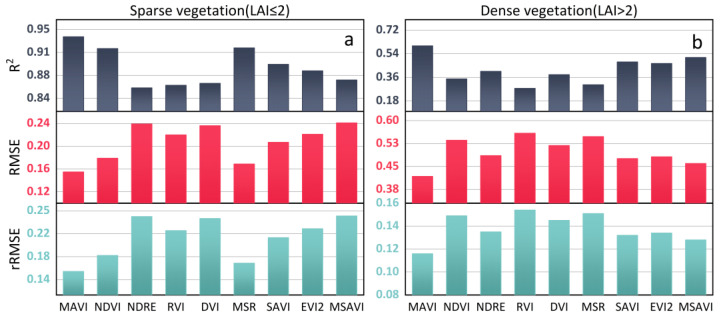
Fitting accuracy of measured LAI and VI with (**a**) Sparse and (**b**) dense vegetation (the VIs are from Table 3).

To further evaluate the MAVI’s performance, we compared the empirical relationship between VI and LAI at different vegetation densities, as presented in Table 4 and Figure 11. The results indicate that the prediction accuracy of the MAVI, the MSR, and the NDVI for LAI in sparse vegetation is similar, and higher than of other VIs. But on the whole, the MAVI exhibits the highest prediction accuracy (R^2^ = 0.934, RMSE = 0.155, rRMSE = 0.157), suggesting it has the strongest correlation with the LAI and better tracks LAI changes in sparse vegetation. The prediction accuracy of the SAVI, the EVI2, the RVI, the DVI, the NDRE, and the MSAVI gradually decreases, with the MSAVI having the lowest prediction accuracy (R^2^ = 0.868, RMSE = 0.241, rRMSE = 0.244). This result indicates that although VIs like the SAVI and the EVI2, etc., exhibit resistance to different soil reflectance conditions, they may still be influenced by other vegetation parameters (C_m_, N, etc.). Moreover, when the LAI > 2, the NDVI’s retrieval performance is poor, revealing that the NDVI’s saturation effect in dense vegetation significantly reduced the estimation accuracy of LAI. The NDRE, the SAVI, the EVI2, the DVI, the and MSAVI all offer higher accuracy for the LAI than the NDVI, indicating they have the ability to mitigate the saturation effect on LAI estimation. The RVI and the MSR perform poorly in LAI estimation, indicating less than ideal interference resistance. Among all the vegetation indices, the MAVI demonstrates the highest estimation accuracy (R^2^ = 0.601, RMSE = 0.418, rRMSE = 0.116) for LAI in dense vegetation, indicating that multi-angular observation of the tower-based platform is helpful for improving the accuracy of LAI retrieval. The MAVI based on the multi-angular observation has achieved excellent performance, which shows that reasonable utilization of the multi-angular reflectance can effectively improve the accuracy of LAI estimation at the site scale. This innovative approach expanded the application of multi-angular observation.

## 4. Discussion

### 4.1. Analysis of Multi-Angular Spectral Observations

Numerous studies have shown that single-angular reflectance cannot effectively reflect the three-dimensional information of vegetation, so more and more scholars are attempting to use multi-angular reflectance to retrieval LAI [5,28,72]. However, there are few studies on mining and utilizing multi-angular reflectance information of SZA changes. To solve this problem, this study aims to propose a method that utilizes rich multi-angular information of SZA changes, so as to better estimate LAI.

To enhance the accuracy of LAI retrieval, researchers have proposed various methods. For instance, researchers [65] replaced the red band of the NDVI with the red-edge band and proposed the red-edge NDVI, which can be applied to estimate LAI. However, the red-edge band is highly sensitive to changes in the LCC, which can complicate LAI retrieval. To address this concern, Zhang et al. [63] proposed the CIVI, which incorporates the red-edge band while mitigating the sensitivity of the vegetation index to the LCC, enabling LAI retrieval. Nonetheless, the CIVI’s formula is complex and susceptible to variations in specific parameters such as N and C_m_. In an effort to minimize soil influence on canopy spectra, Huete et al. [70] developed the SAVI by introducing the soil adjustment factor L into the VI formula. However, determining the actual value of the adjustment factor L remains challenging, posing a major hurdle for SAVI adoption, as the value of 0.5 may not be suitable for all scenarios. Similar improved VIs such as the EVI2 and the MSAVI, which retrieve LAI without the trouble of saturation effects and are less sensitive to soil background, have also been proposed [69,71]. However, as illustrated in Figure 9, they exhibit high sensitivity to crucial parameters like C_m_ and N, etc. The MSR and the WDRVI were introduced to address the saturation effect of the NDVI but do not account for the influence of other vegetation parameters [17,68]. Multi-angular observation has been recognized as an effective means to enhance LAI retrieval accuracy. Hasegawa et al. [5] developed the NHVI using multi-angular observation data but overlooked the contribution of soil background. Vegetation indices such as the HDDI, the NDVIhd, and the NDVIHs, which are based on hotspot and darkspot characteristics and can describe canopy structure, have been proposed but are limited in many vegetation scenarios [27,28].

In recent years, scholars have established numerous automatic spectral observation systems installed on tower-based platforms for long-term continuous observation of canopy spectra across various vegetation types [31,34,38]. The observation data collected from tower-based platforms can be used to explore the relationship between spectral signals and flux data, providing ground truth data for satellite remote sensing validation [31]. The LAI is a critical parameter with implications for hydrology, ecology, and climate modeling [3,73]. Thus, precise LAI retrieval at the tower-based site scale holds significant ecological research value. In tower-based spectral observations, the VZA is typically fixed while the SZA varies throughout the day. This observation method maintains a fixed proportion of canopy and background within the observation field, but the proportion of light and shadow changes. In practical observations, the illuminated ratio within the FOV increases as the SZA decreases. At the same time, the soil reflectance contributes more and the vegetation information is more, so the reflectance is more sensitive to the soil background and the LAI. Conversely, reflectance sensitivity to the soil background and the LAI decreases as the SZA rises. Based on this principle, we proposed the MAVI, which has the potential to reduce NDVI’s sensitivity to soil while improving its respond to LAI.

### 4.2. The Advantages and Applications of the MAVI

The proposed MAVI, which rationally combines reflectance from different SZAs, offers several advantages. Firstly, as shown in Figure 6 and Figure 7, the MAVI effectively reduced sensitivity to soil reflectance while eliminating the saturation effect of vegetation indices on the LAI. The relationship between VI and LAI is strengthened, making it more robust. Secondly, the MAVI strengthened the VI–LAI relationship while retaining the NDVI’s advantage of insensitivity to critical parameters such as C_m_, LCC, and N. Thirdly, the MAVI boasts a straightforward structure, minimizing uncertainty compared to complex formulas. Finally, PROSAIL generates simulation data with universal applicability, and the MAVI is proposed based on PROSAIL simulation data. Therefore, the MAVI, calculated based on PROSAIL simulation data, holds potential for LAI estimation across various vegetation conditions. The superior performance of the MAVI in LAI retrieval for different vegetation densities supports this inference. Overall, the MAVI effectively harnessed the rich information from multi-angular reflectance in tower-based observations, and successfully achieved high-precision LAI retrieval by mitigating soil and saturation interference.

As a novel vegetation index, the MAVI is of great significance for LAI retrieval. Firstly, the MAVI provided a simple but effective solution to mine the information of multi-angular reflectance of SZA variations. Secondly, the MAVI method can effectively overcome the shortcomings of using the NDVI to retrieve LAI. At the same time, there are numerous tower-based observation platforms all over the world, and the MAVI method can be extended to the LAI retrieval at various sites, which can effectively improve the prediction accuracy of LAI and better serve the satellite verification. Finally, the MAVI made use of the information from varying SZAs and a fixed VZA, which can avoid changing the field of view, and can be better applied to observation on small plots.

### 4.3. Limitations and Future Prospects

However, the MAVI also has some limitations. Firstly, as mentioned in Section 3.3, the MAVI is also sensitive to LAD, likely because multi-angular reflectance has the ability to characterize vegetation canopy structure. Given the intricate influence of vegetation parameters on reflectance and the limited spectral band obtainable from the spectrometer on the tower-based platform, developing a VI solely sensitive to target parameters is highly impractical [63]. At the same time, the observation object of tower-based remote sensing is generally limited to single species. Therefore, we ignored the interference caused by different plant types in this study. Secondly, as illustrated in Figure 8, the MAVI’s SP is lower than that of traditional vegetation indices like the RVI and the DVI, with saturation occurring when the LAI reaches 4.8. However, considering that the observation primarily pertains to maize and measurements indicate the LAI of maize at the DM site typically falls below 4.5, the MAVI remains suitable for tower-based LAI retrieval. Finally, this study only acquired observation data for maize, and despite three years of observation the sample size remains relatively small. Future research should aim to validate these results with a larger number of samples and more measurements of different vegetation types.

## 5. Conclusions

Multi-angular observation has the potential to provide three-dimensional vegetation information and enhance LAI estimation accuracy. In this paper, by analyzing the reflectance changes of different SZAs, it was found that with the increase in SZA, the sensitivity of NIR to LAI and soil gradually decreased. Based on this principle, a novel multi-angular VI, the MAVI, is proposed. Results demonstrate that the MAVI exhibited high sensitivity to the LAI and effectively mitigated interference from soil background reflectance in LAI retrieval. This study assessed the MAVI’s performance using measurements at the site and comparing it with other VIs, including the NDVI, the NDRE, the RVI, the DVI, the MSR, the SAVI, the MSAVI, and the EVI2. Validation results indicate that the MAVI can successfully estimate LAI in both sparse and dense vegetation. This research underscored the importance of considering multi-angular observation in VI-based LAI retrieval.

## Figures and Tables

**Table 1:** The quadrat information for three years.

Year	Quadrat	Plant Spacing (cm)	Row Spacing (cm)	Ridge Spacing (cm)	First Watering	Second Watering	Third Watering	Fourth Watering
2018	1	25	35	80	-	14 June 2018	2 July 2018	21 July 2018
	2	25	35	80	-	14 June 2018	2 July 2018	21 July 2018
	3	25	35	80	-	14 June 2018	2 July 2018	21 July 2018
2019	1	-	35	65	16 June 2019	-	-	-
	2	-	35	65	16 June 2019	-	-	-
	3	-	35	65	16 June 2019	-	-	-
2020	1	-	120	20	-	-	30 July 2020	-
	2	-	110	25	-	-	30 July 2020	-
	3	-	100	23	-	-	30 July 2020	-

**Table 2:** The setting of parameters in the PROSAIL model.

Parameter	Parameter Description	Unit	Fixed Value	Range	References
Input parameters of PROSPECT model					
C_ab_	Chlorophyll content	μg/cm^2^	40	20–80	[52]
C_ar_	Carotenoid content	μg/cm^2^	8	4–16	[52]
C_w_	Equivalent water thickness	cm	0.015	0.015	[53]
C_m_	Dry matter content	g/cm^2^	0.004	0.004–0.02	[54]
N	Leaf structure coefficient	-	1.5	1–2	[54]
Input parameters of SAIL model					
LAI	Leaf area index	m^2^/m^2^	1	0.2–5	[55]
LAD	Leaf inclination angle distribution	-	−0.35	[1,0], [0,−1], [0,1], [−0.35,−0.15]	[56]
		-	−0.15		[56]
hspot	Hotspot factor	-	0.1	0.1	[57]
Ps	Soil brightness coefficient	-	0.2	0–1	[54]
Sky1	Sky scattered light ratio	-	0.847	0.847	[3]
SZA	Solar zenith angle	degree	20	0–60	Measured
VZA	Viewing zenith angle	degree	25	25	Measured
RAA	Relative azimuth angle	degree	60	60, 130	Measured

[1,0], planophile; [0,−1], plagiophile; [0,1], extremophile; [−0.35,−0.15], spherical.

**Table 3:** Multispectral indices which are employed for retrieving LAI.

Index	Definition	Reference
NDVI (normalized difference vegetation index)	$NDVI=\frac{\rho_{800}-\rho_{680}}{\rho_{800}+\rho_{680}}$	Rouse et al. [64]
NDRE (normalized difference red edge vegetation index)	$NDRE=\frac{\rho_{800}-\rho_{720}}{\rho_{800}+\rho_{720}}$	Gitelson et al. [65]
RVI (ratio vegetation index)	$RVI=\rho_{800}/\rho_{680}$	Broge et al. [67]
DVI (difference vegetation index)	$DVI=\rho_{800}-\rho_{680}$	Richardson et al. [66]
MSR (modified simple ratio vegetation index)	$MSR=\frac{\rho_{800}/\rho_{680}-1}{\sqrt{\rho_{800}/\rho_{680}+1}}$	Haboudane et al. [68]
EVI2 (enhanced vegetation index 2)	$EVI2=\frac{2.5*\left(\rho_{800}-\rho_{680}\right)}{\left(\rho_{800}+2.4*\rho_{680}+1\right)}$	Jiang et al. [69]
SAVI (soil-adjusted vegetation index)	$SAVI=1.5*\frac{\rho_{800}-\rho_{680}}{\left(\rho_{800}+\rho_{680}+0.5\right)}$	Huete [70]
MSAVI (modified soil-adjusted vegetation index)	MSAVI=0.5∗(2∗ρ800+1−((2∗ρ800+1)^2−8∗(ρ800−ρ680 ))^0.5)	Qi et al. [71]

**Table 4:** Evaluation of LAI retrieval accuracy with sparse (0 < LAI ≤ 2) and dense (LAI > 2) vegetation over the maize canopy based on measured data (the VIs are introduced in Table 3).

	LAI ≤ 2				LAI > 2			
	Fit Model	R^2^	RMSE	rRMSE	Fit Model	R^2^	RMSE	rRMSE
MAVI	y = 0.094*e^4.747*x^	0.934	0.155	0.157	y = 0.220*e^3.715*x^	0.601	0.418	0.116
NDVI	y = 0.129*e^3.247*x^	0.916	0.179	0.182	y = 0.011*e^6.304*x^	0.349	0.536	0.149
NDRE	y = 0.171*e^4.953*x^	0.856	0.239	0.243	y = 0.345*e^3.832*x^	0.406	0.486	0.135
RVI	y = 0.211 + 0.155*x	0.860	0.220	0.221	y = 2.352 + 0.055*x	0.276	0.559	0.154
DVI	y = 0.120*e^9.784*x^	0.863	0.236	0.240	y = 0.950*e^3.505*x^	0.381	0.519	0.145
MSR	y = 0.105 + 0.611*x	0.917	0.169	0.170	y = 1.182 + 0.550*x	0.304	0.548	0.151
SAVI	y = 0.123*e^5.449*x^	0.892	0.207	0.210	y = 0.321*e^3.892*x^	0.478	0.476	0.132
EVI2	y = 0.140*e^5.142*x^	0.882	0.221	0.224	y = 0.521*e^2.939*x^	0.466	0.482	0.134
MSAVI	y = 0.151*e^5.096*x^	0.868	0.241	0.244	y = 0.610*e^2.608*x^	0.512	0.460	0.128

## Data Availability

Due to the nature of this research, participants of this study did not agree for their data to be shared publicly, so supporting data is not available.
